# Serial prophylactic exchange blood transfusion in pregnant women with sickle cell disease (TAPS-2): study protocol for a randomised controlled feasibility trial

**DOI:** 10.1186/s13063-020-4212-8

**Published:** 2020-04-20

**Authors:** Laura L. Oakley, Moji Awogbade, Sarah Brien, Annette Briley, Maria Chorozoglou, Emma Drasar, Jemma Johns, Elizabeth Rhodes, Vicky Robinson, Paul Seed, Joseph Sharif, Claire Singh, Paul Telfer, Hilary Thompson, Ingrid Watt-Coote, Jo Howard, Eugene Oteng-Ntim

**Affiliations:** 1 Department of Non-communicable Disease Epidemiology, London School of Hygiene and Tropical Medicine, Keppel Street, London, WC1E 7HT UK; 2 Centre for Fertility and Health, Norwegian Institute of Public Health, P.O. Box 222, Skøyen, N-0213 Oslo, Norway; 3 King’s College Hospital NHS Foundation Trust, Denmark Hill, London, SE5 9RS UK; 4 Primary Care, Population Sciences and Medical Education, Faculty of Medicine, University of Southampton, Southampton General Hospital, Southampton, SO16 6YD UK; 5 Guy’s and St Thomas’ NHS Foundation Trust, Westminster Bridge Road, London, SE1 7EH UK; 6 Caring Futures Institute, College of Nursing and Health Sciences, Flinders University, Adelaide, 5001 South Australia Australia; 7 Southampton Health Technology Assessment Centre, Faculty of Medicine, University of Southampton, The University of Southampton Science Park, Alpha House, Enterprise Road, Southampton, SO17 1BJ UK; 8 Whittington Health NHS Trust, Magdala Avenue, London, N19 5NF UK; 9 St George’s University Hospitals NHS Foundation Trust, Blackshaw Road, Tooting, London, SW17 0QT UK; 10 Division of Women’s Health, Kings College London, St Thomas’ Hospital, 10th floor North Wing, Lambeth Palace Road, London, SE1 7EH UK; 11 Manchester University NHS Foundation Trust, Oxford Road, Manchester, M13 9WL UK; 12 Barts Health NHS Trust, 80 Newark Street, London, E1 2ES UK; 13 Department of Haematological Medicine, King’s College London, Rayne Institute, London, SE5 9NU UK

**Keywords:** Sickle cell disease, Pregnancy, Blood transfusion, Feasibility, Randomised controlled trial, Economic evaluation, Qualitative

## Abstract

**Background:**

Pregnancies in women with sickle cell disease (SCD) are associated with a higher risk of sickle and pregnancy complications. Limited options exist for treating SCD during pregnancy. Serial prophylactic exchange blood transfusion (SPEBT) has been shown to be effective in treating SCD outside pregnancy, but evidence is lacking regarding its use during pregnancy. The aim of this study is to assess the feasibility and acceptability of conducting a future phase 3 randomised controlled trial (RCT) to establish the clinical and cost effectiveness of SPEBT in pregnant women with SCD.

**Methods:**

The study is an individually randomised, two-arm, feasibility trial with embedded qualitative and health economic studies. Fifty women, 18 years of age and older, with SCD and a singleton pregnancy at ≤ 18 weeks’ gestation will be recruited from six hospitals in England. Randomisation will be conducted using a secure online database and minimised by centre, SCD genotype and maternal age. Women allocated to the intervention arm will receive SPEBT commencing at ≤ 18 weeks’ gestation, performed using automated erythrocytapheresis every 6–10 weeks until the end of pregnancy, aiming to maintain HbS% or combined HbS/HbC% below 30%. Women in the standard care arm will only receive transfusion when clinically indicated. The primary outcome will be the recruitment rate. Additional endpoints include reasons for refusal to participate, attrition rate, protocol adherence, and maternal and neonatal outcomes. Women will be monitored throughout pregnancy to assess maternal, sickle, and foetal complications. Detailed information about adverse events (including hospital admission) and birth outcomes will be extracted from medical records and via interview at 6 weeks postpartum. An embedded qualitative study will consist of interviews with (a) 15–25 trial participants to assess experiences and acceptability, (b) 5–15 women who decline to participate to identify barriers to recruitment and (c) 15–20 clinical staff to explore fidelity and acceptability. A health economic study will inform a future cost effectiveness and cost-utility analysis.

**Discussion:**

This feasibility study aims to rigorously evaluate SPEBT as a treatment for SCD in pregnancy and its impact on maternal and infant outcomes.

**Trial registration:**

NIH registry (www.clinicaltrials.gov), registration number NCT03975894 (registered 05/06/19); ISRCTN (www.isrctn.com), registration number ISRCTN52684446 (retrospectively registered 02/08/19).

## Background

Sickle Cell Disease (SCD) is one of the most common inherited diseases worldwide [1]. It is characterised by anaemia, intermittent unpredictable episodes of severe pain (vaso-occlusive crisis) and chronic complications including stroke, retinopathy, chronic lung disease, pulmonary hypertension and renal dysfunction [2]. Pregnancy in women with SCD is associated with an increased risk of sickle-related complications (pain crises, pulmonary complications, infection), as well as a higher risk of maternal and perinatal mortality, pregnancy-related complications (proteinuric hypertension, venous thrombosis, caesarean delivery) and perinatal complications (intrauterine growth restriction, preterm birth and low birthweight) [3, 4]. More than 300,000 children are born with the disease annually worldwide [5], most in sub-Saharan Africa. In 2016–17, 274 infants with SCD were born in the United Kingdom [6]. In countries with advanced medical care, most children will survive to adulthood [1], with good expectation of having their own families. In the United Kingdom, an estimated 15,000 individuals live with SCD, with approximately 110 pregnancies in women with SCD each year [3]. Despite obstetric interventions and improved supportive care, these pregnancies have high rates of complications, with a persisting relative risk of maternal mortality of 14.26 (95% CI 7.52–27.07) [4]. Pregnancy in women with SCD is also associated with recurrent hospital and critical care admissions with inherent associated costs [3]. Currently, only two disease-modifying treatments are available for patients with SCD: hydroxycarbamide and blood transfusion. The former has a risk of teratogenicity and is not recommended during pregnancy [7]. Blood transfusion is an important treatment in SCD and, outside pregnancy, has proven efficacy in the treatment of acute sickle complications and for the prevention of pain, acute chest syndrome and neurological complications [1]. Blood transfusion can be given as a simple or ‘top-up’ transfusion, which will improve haemoglobin (Hb) levels and oxygen carriage and offer a moderate decrease in red cells containing sickle Hb. The alternative approach is exchange transfusion, i.e., the sequential removal of patient red blood cells and replacement with donor red cells; this produces a more marked reduction in red blood cells containing sickle Hb. This can be undertaken manually or using automated apheresis. Automated serial prophylactic exchange blood transfusion (SPEBT) is the preferred mechanism of long-term transfusion therapy. SPEBT has proven results in improving clinical and cost effectiveness, as it is required less frequently than simple or manual exchange transfusion and results in better control of the haemoglobin, sickle percentage and reduced iron loading [8]. During pregnancy, current clinical care requires that transfusion be administered for emergency indications, such as for symptomatic anaemia or acute chest syndrome [7].

### Summary of findings from previous clinical trials and systematic reviews

The role of blood transfusion in pregnant women with SCD was investigated by a Cochrane review in 2016 [9]. This review identified one US trial published in 1988 involving 72 women randomised to serial prophylactic transfusion versus standard care (transfusion for clinical indications only) [10]. The authors reported decreased pain episodes in the group receiving serial prophylactic transfusion, and whilst it detected no differences in other maternal and neonatal complications, it was underpowered to do so [10]. Women were enrolled up to 28 weeks’ gestation, with a mean time of first transfusion of 14 weeks, and patients were treated with serial simple or partial exchange transfusion to maintain Hb between 100 and 110 g/dl and HbS% below 35%. The Cochrane review concluded that insufficient evidence existed to provide reliable advice for an optimal blood transfusion policy for pregnant women with SCD and recommended a more rigorous randomised controlled trial (RCT) to address this question. Another systematic review and meta-analysis published in 2015 [11] identified 11 cohort studies, in addition to the RCT described above. Meta-analysis of these studies showed that prophylactic transfusion was associated with a reduction in maternal mortality (OR 0.23; 95% CI 0.06–0.91), vaso-occlusive pain episodes (OR 0.26, 95% CI 0.09–0.76), pulmonary complications (OR 0.25, 95% CI 0.09–0.72), preterm birth (OR 0.59, 95% CI 0.37–0.96) and perinatal mortality (OR 0.43, 95% CI 0.19–0.99). Overall event rates were low, and the studies had a high risk of bias because of confounding and low event size; therefore, the benefits of prophylactic transfusion may have been overestimated. The authors concluded that a multicentre RCT is needed to determine whether the potential benefits of SPEBT outweigh the associated risks. We have identified one further study published since this systematic review: an observational study, comparing outcomes in 24 pregnancies in women with HbSC for whom 10 received SPEBT and 14 received standard care [12]. The standard care group experienced higher frequencies of SCD-related complications (36% vs 10%), and these complications were more severe. All cases of acute chest syndrome (*n* = 4) occurred in the standard care group. A statistically significant difference was observed in the gestational age at birth (38.7 weeks in the transfusion group vs 34.4 weeks in standard care), and a higher frequency of preterm births was observed in the standard care group (69% vs 30%). The authors concluded that a reduction of adverse outcomes occurred in the patients who received SPEBT, although the study was limited by a small sample size.

Significant knowledge gaps exist regarding the effectiveness of SPEBT for pregnant women with SCD [9, 11], and a recent survey of 90 clinicians providing care to pregnant women with SCD reported that 94% (*n* = 85) supported the need for a RCT to assess the benefits of SPEBT in this population [13]. A detailed search of trial registries including UKCRN, ISRCTN, Clinical Trials.gov and European Union Clinical Trial registry showed no registered current trials on transfusion in pregnant women with SCD. The proposed research, designed in conjunction with PPI representatives, clinical staff and other stakeholders, has the potential to improve the evidence base regarding the management of pregnant women with SCD.

## Methods/Design

### Aim

The aim of the current study is to assess the feasibility and acceptability of conducting an RCT to establish the clinical and cost effectiveness of serial prophylactic exchange blood transfusion (SPEBT) in pregnant women with SCD. The detailed objectives of the study are shown in Table 1.

**Table 1:** Detailed study objectives by sub-study

		Objectives
**Trial**	**Feasibility: primary**	1. Assess the willingness of pregnant women with SCD to take part in a randomised controlled trial comparing SPEBT to standard care.
	**Feasibility: secondary**	2. Identify barriers and facilitators to participation in the trial, including assessing reasons for refusal. 3. Assess retention rates of participants throughout pregnancy in both arms of the study. 4. Assess the willingness of clinicians to recruit into this trial.
	**Clinical: infant**	5. Assess the proportion from the control arm advised clinically to start prophylactic blood transfusion. 6. Measure clinical outcomes for women and infants including an initial preliminary assessment of efficacy for future definitive trial. 7. Generate data to inform the design of a definitive trial, including identifying the primary outcome.
	**Clinical: maternal**	
	**Safety**	8. Record safety issues around blood transfusions in both arms of the study
**Qualitative**	**Feasibility**	9. Identify barriers and facilitators to participation from study participants, clinicians and, where possible, those unwilling to participate 10. Identify strategies to optimise recruitment and retention 11. Assess acceptability of the intervention, trial procedures and study conduct, including identifying the core outcomes that are considered important to measure and preference for either HRQOL measure 12. Identify reasons for attrition 13. Assess women’s experience of taking part in the study
**Economic**	**Resource Use & Costs**	14. Explore the cost implications of the proposed intervention and to assess measurement tools and methods
	**HRQoL & health benefits**	15. Assess two widely used HRQoL measures against each other and other health benefits

### Design and setting

This is a UK, multicentre, randomised, two-arm, feasibility trial with embedded qualitative and economic studies.

### Participants

The participants are women ≥18 years of age with SCD (all genotypes, with confirmatory laboratory results) and a singleton pregnancy at < 18^+ 0^ weeks’ gestation.

#### Exclusion criteria

Women will be ineligible if any of the following criteria apply:

- Unable or unwilling to give written informed consent
- On long-term transfusion programme prior to pregnancy for the amelioration of SCD
- Unable to receive blood transfusion for social, clinical or religious reasons
- Current diagnosis of major medical or psychiatric comorbidity that, in the randomising clinicians’ opinion, renders them unable to enter the trial
- Prior hyperhaemolysis
- Red cell phenotype or antibodies present prevent likely provision of adequate red cell units to support elective EBT programme

### Participant identification and recruitment

**Fig. 1:**
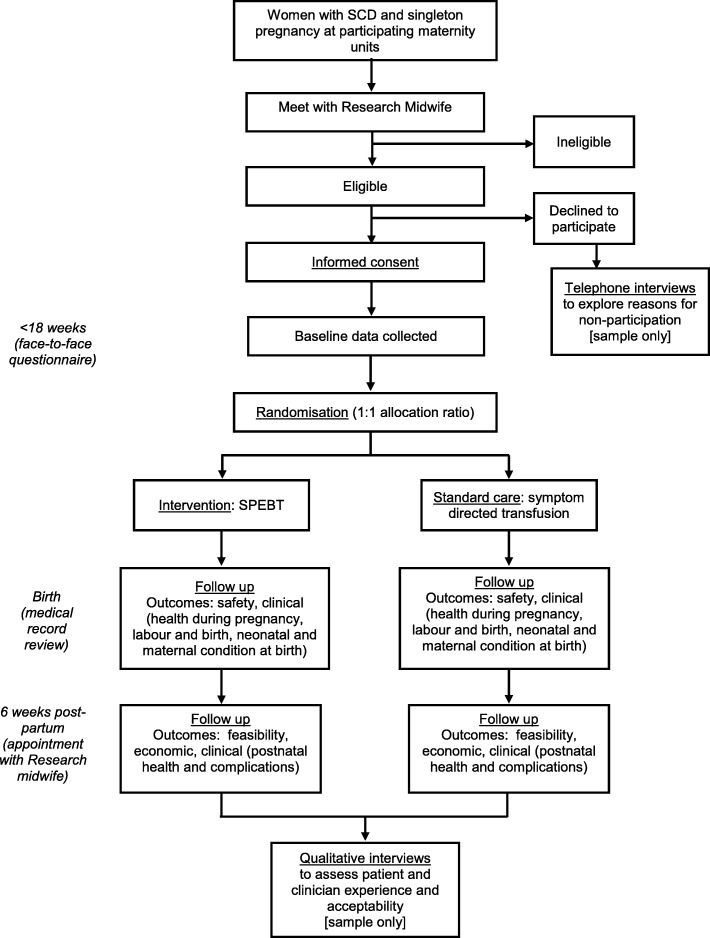
Study flowchart

**Fig. 2:**
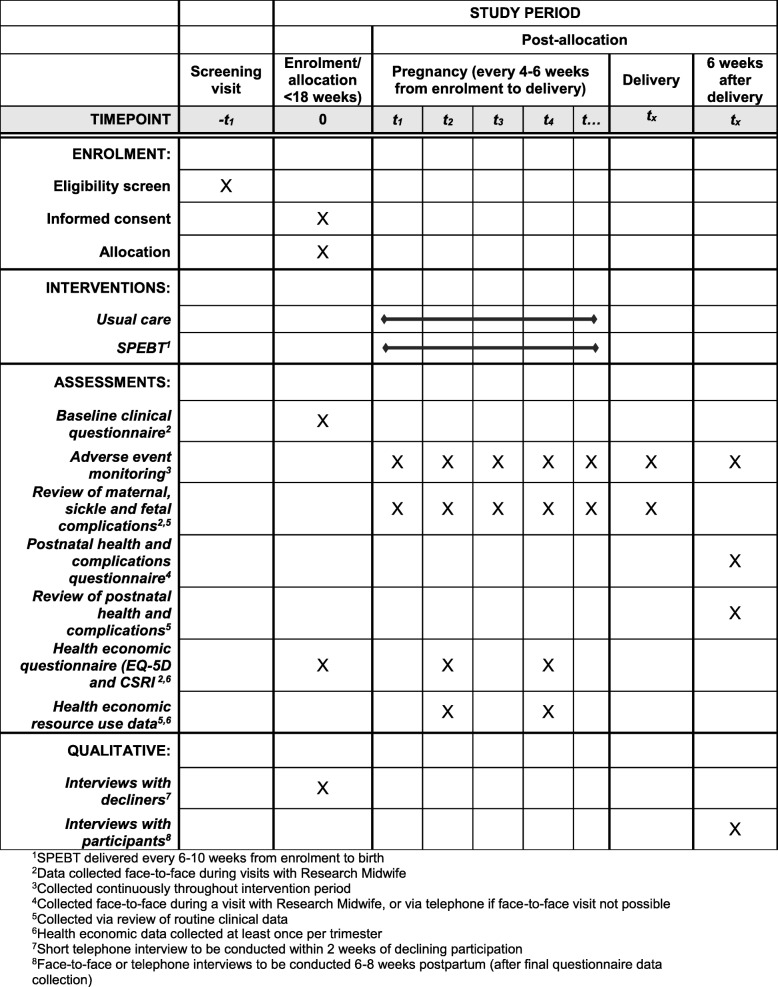
Schedule of enrolment, interventions, and assessments for TAPS2

Potentially eligible women with SCD will receive a verbal explanation of the study and will be given the Patient Information Sheet (PIS) at either the SCD clinic or at the first antenatal clinic appointment at participating hospitals (Fig. 1). Women who are interested in participating will be invited to meet the research midwife prior to 18^+ 0^ weeks’ gestation to fully assess eligibility and answer any queries. If the woman decides to participate, written informed consent will be obtained by one of the trial physicians on the study delegation log. Participants have the right to withdraw at any time without giving a reason. The number of women approached, eligible and declining participation (when first approached or at consent), and the reasons for non-participation will be collected via the secure Internet-based study database (MedSciNet™). The schedule of procedures for the study is presented in Fig. 2.

### Randomisation

Randomisation will be achieved via the study-specific Internet-based secure data management system (MedSciNet™), which is the repository for all trial data. The unit of randomisation will be the individual participant, allocated in a ratio of 1:1 to intervention and standard care. Participants will be randomised using minimisation, balancing on centre, SCD genotype and maternal age. Participants will be present at the time of randomisation and will be informed of their allocation. Blinding of the participants or the attending care teams will not be possible due to the nature of the intervention.

### Standard care

All participants will receive routine NHS antenatal care for pregnant women with SCD based on the NICE accredited RCOG Guidelines [7] and in accordance with local policies in the participating units. This will include multidisciplinary care, involving obstetricians, haematologists and midwives, prophylactic folic acid to improve anaemia, penicillin to reduce the risk of infection and low-dose aspirin. Women in the standard care group will only receive a blood transfusion where clinically indicated.

### Intervention

Women allocated to the intervention will receive SPEBT starting between 6^+ 0^–18^+ 0^ weeks of gestation. SPEBT will be performed using automated apheresis technology, approximately every 6–10 weeks until the end of pregnancy, aiming to maintain HbS% below 30% or combined HbS and HbC below 30%. The procedure will be carried out in accordance with local practices on the haematology day unit or the antenatal day ward by the clinical or research nurse/midwife, haematology day unit staff or specialist sickle nursing staff, using standard operating procedures. Where peripheral venous access is not possible, femoral line access will be used. Blood tests (full blood count, HbS%, group and screen) will be carried out 1–3 days prior to the procedure and immediately post-procedure in accordance with local guidelines and practice. The number of red cell units used per transfusion will depend on the patient weight and pre-transfusion HbS% but will usually be between 6 and 8 units on each occasion of transfusion.

### Data collection

All baseline and follow-up data will be entered on the secure internet-based data management system contemporaneously, with the exception of pregnancy outcome follow-up, which will be updated postnatally. Additional data will be extracted from NHS medical records and entered directly onto the system by members of the research team. This will negate the need for paper clinical research files and will minimise errors in transcription, whilst facilitating expedient data monitoring and cleaning. Once data collection is complete, the trial team at the lead centre (GSTT) will have access to the final trial dataset, and analyses will be led by the appointed trial statistician.

At baseline, information will be collected on previous medical and obstetric history, current pregnancy, socio-demographic information and quality of life (QOL) data. All participants will be seen every 4 to 6 weeks during pregnancy at the specialist sickle–obstetric clinic or the high-risk obstetric clinic to assess and record maternal, sickle and foetal complications. Health economic data will be collected at least once per trimester using the 3 L and 5 L Euroquol EQ-5D questionnaires [14]. Following the birth, detailed information regarding health in late pregnancy, labour and birth, postnatal complications, admissions to higher levels of care and length of maternal hospital stay will be extracted from NHS medical records. Neonatal data will include the need for resuscitation, Apgar scores, admission to NICU/SCBU, feeding method and duration of hospital stay (in each level of care). At 6 weeks postpartum, participants will be invited to an appointment with the research team to provide information about their and their infant’s postpartum complications and health. QoL data will also be collected. If a visit is not feasible, a telephone interview will be conducted. A subset of study participants will be interviewed to assess views and experiences of trial participation. Postnatal medical records will also be reviewed if available.

### Outcomes

#### Primary outcome

The primary outcome is the efficacy of recruitment strategy, measured by comparing the number of recruited and randomised women with the total number of eligible women in all centres.

#### Secondary outcomes

We will measure the following secondary feasibility outcomes:

- The number of women screened who meet the study eligibility criteria
- Reasons for eligible women declining participation
- Rate and reasons for attrition (monitored by assessing the number of women completing the study, and by reporting reasons given (if any) for study drop-out)
- Protocol adherence (measured by the number of women allocated to the intervention group who receive SPEBT as intended (beginning < 18 weeks, transfusions every 6–10 weeks, to maintain a HbS < 30% or combined HbS & HbC < 30%), and the number of women in the standard care group who receive a transfusion due to clinical need)
- Patient experience and acceptability (assessed through qualitative methods)
- Views and experiences of clinical staff caring for women in both arms of the trial (assessed through qualitative methods)

The following clinical outcomes will also be measured:

- Neonatal: Foetal demise/stillbirth/neonatal death, Apgar score at 5 min, birthweight, gestation at birth, NICU/SCBU admission with reason and length of stay, feeding method at discharge
- Maternal: Antenatal hospital admissions and critical care admissions, length of stay (total inpatient, antenatal and postnatal nights), frequency and severity of painful crisis (severity of crisis will be measured as mild, moderate, severe or extremely severe, where mild crises may or may not have required pain medication but did not prevent normal activity, moderate crises required medication and caused significant changes in daily activities, severe crises required attendance at hospital, and extremely severe required hospital admission); use of opioid analgesics (excluding labour); frequency of SCD-related complications (acute chest syndrome, pre-eclampsia); number and reason for any transfusion (in the standard care arm); number of transfusions (in the intervention arm), mode of birth, and estimated blood loss; postnatal health and complications, including pain and primary and secondary postpartum haemorrhage; and hospital readmission after postnatal discharge, thromboembolism, infection, or depression

We will additionally collect data on the following safety outcomes: transfusion reactions, alloimmunisation and delayed haemolytic transfusion reaction.

### Health economic sub-study

Alongside this feasibility RCT, we will conduct a health economic sub-study to inform a future cost effectiveness and will conduct a cost-utility analysis within our definitive RCT. We will collect data using two preference-based QoL measures, with the aim of identifying the most suitable HRQoL instrument. We plan to address the following research questions: what are the cost implications of SPEBT in pregnant women with SCD (comparison of costs of both treatment options compared to widely-used NHS Reference Costs), and, what are the implications of using differing cost methods when assessing the cost effectiveness of the intervention?

### Nested qualitative study

A nested qualitative study aims to capture the views and experiences of both participants and clinical staff involved in the study to inform the design of a definitive trial. Face-to-face semi-structured interviews will be conducted postnatally with 15–25 women recruited to both study arms; telephone interviews will be conducted if face-to-face interviews are not possible. Participants will be purposively sampled, using a maximum variation sampling approach on key characteristics (e.g., those who completed versus those who dropped out, across different recruitment sites). Interviews will explore the acceptability of randomisation and study conduct (trial appointments, measurements and blood tests and recruitment/retention). In addition, participants in the intervention arm will be asked for their perception and experience of SPEBT (e.g., timings, regime, adverse effects) and the impact of the intervention on their daily life. We will also conduct 5–15 short telephone interviews with women who declined participation in the study. These interviews will explore barriers to participation, helping to identify strategies to enhance recruitment in a future trial. In addition, interviews with 15–20 clinical staff considered ‘key informants’ (e.g., research nurses/midwives, sickle nurse specialists, sickle haematologists and sickle obstetricians in each centre) will explore fidelity and acceptability. Written informed consent for the qualitative study for trial participants will be obtained prior to contact by the researcher, and consent will be reaffirmed immediately prior to interview. For clinicians and for those women who were approached but who did not take part in the trial, written informed consent will be obtained before the interview commences. All interviews will be digitally recorded verbatim, transcribed and analysed using thematic analysis within and across individual interviews, using the constant comparison approach to identify themes and subthemes. Standard methods will be employed to ensure rigour (e.g., audit trail, reflexive diaries, double coding etc). We will follow recent guidance regarding the effective use of qualitative research in feasibility studies for RCTs [15].

### Trial withdrawal or discontinuation

We will withdraw participants if they lack the capacity to provide ongoing consent. Participants have the right to withdraw from the study at any time, without giving a reason. The PI and local investigators will also have the right to withdraw patients from the study intervention in the event of inter-current illness, AEs, SAEs, SUSARs, protocol violations, administrative reasons or other reasons. Women who wish to withdraw from the trial will be asked to confirm whether they are still willing to provide routine follow-up data and/or participate in interviews (assessed as per intention to treat).

The trial may be prematurely discontinued by the Sponsor, Chief Investigator or Regulatory Authority on the basis of new safety information or for other reasons given by the Trial Steering Committee (TSC), regulatory authority or ethics committee concerned. If the trial is prematurely discontinued, active participants will be informed, and no further participant data will be collected. The Competent Authority and Research Ethics Committee will be informed within 15 days of the early termination of the trial.

### Power and sample size

The study is designed to establish the rates at which women with SCD can be recruited and retained in a future definitive RCT. A sample of 40 women (20 in each arm) will allow us to estimate the overall recruitment rate per woman with SCD to within 10% of the true value. This approach will accurately predict the recruitment rate in the main study and determine the required length of the recruitment period.

Over an 18-month recruitment period, we estimate that a total of approximately 100 women with SCD will be seen in participating maternity units. Assuming a 50–60% recruitment rate (allowing for ineligible women and those who do not wish to take part), we plan to recruit 50 participants during the study period (25 in each arm). Allowing for a 20% loss to follow-up, we estimate that 40 women should complete postpartum follow-up, consistent with our required sample size.

### Analysis

Our primary outcome (overall recruitment rate as a percentage of eligible women) will be estimated using the Clopper-Pearson exact Binomial method for a 95% confidence interval. The number of women screened, eligible, consented, randomised and withdrawn from the study will be reported by site, and overall numbers summarised as a CONSORT flow diagram. Reasons for exclusion and for withdrawal will be summarised. Descriptive statistics including 95% confidence intervals will be presented for all baseline data and clinical outcomes, with a focus on estimates of standard deviation necessary to perform sample size calculations for a future trial. Although the study is expected to be underpowered for clinical outcomes, differences in clinical outcomes will be presented as an initial assessment of efficacy and safety of this treatment, and for inclusion in any future meta-analysis. All analyses will be based on the intention-to-treat (ITT) principle. The trial will be reported in accordance with the CONSORT guidelines on randomised feasibility studies [16].

### Ethical considerations and confidentiality

The trial will be conducted in compliance with the principles of the Declaration of Helsinki (1996), the principles of GCP and in accordance with all applicable regulatory requirements including the Research Governance Framework. NHS ethics approval has been obtained for all centres from the London and Surrey Borders Research Ethics Committee. Written informed consent for trial participants will be obtained by one of the trial physicians, with written informed consent obtained separately for qualitative interviews.

Blood transfusions are regularly used in the clinical care of women with SCD during pregnancy. The most common risk of transfusion is minor reactions such as skin rash or a minor fever, with some reactions (light-headedness, tingling sensation on lips and fingers) specific to the exchange transfusion due to the anticoagulant used. More serious complications include haemolytic transfusion reaction, alloimmunisation and delayed transfusion reaction. We will record all adverse events resulting from the apheresis intervention; for example, issues with venous access and citrate toxicity and serious adverse events (SAEs) will be reported to the Sponsor and REC. Additionally, all participating sites will be expected to follow local and national guidance with respect to the severity of the reaction; this could include but will not be limited to appropriate level and immediacy of medical care, reporting to SHOT (Serious Hazards of Transfusion) and MHRA as well as notification through the local risk management system to enable thorough investigation of the incident.

All trial data will be stored on a secure password-protected study-specific database (MedSciNet™), compliant with GDPR regulations. The database will hold minimal identifiers, with each participant being allocated a unique study ID, and will only be accessible to authorised members of the team. Identifiable data will only be kept in order to facilitate communication with participants. Once women have completed their participation (reached study end or withdrawn), patient identifiable data will be destroyed in accordance with local policies and GDPR.

### Patient and public involvement

In developing the project proposal and protocol, we have worked closely with a user panel of pregnant women with SCD and with the Sickle Cell Society. A PPI group comprising women with SCD has met throughout the development phase and will continue to meet regularly throughout the trial and the dissemination phase. This group will assist with reviewing study documents for accessibility and data interpretation, as well as advising on our dissemination strategy.

### Trial governance and monitoring

A Core Project Management Team (CPMT) consisting of the Chief Investigator, co-investigators and the Project manager will meet monthly to oversee the day-to-day running of the trial. The CPMT will be responsible for regular auditing of trial conduct and for communicating important protocol modifications to the wider project team. The TSC will include experts in the field of maternal medicine and haematology and will meet every 6 months to provide overall supervision of the study, including reviewing progress towards the study milestones and advising the CPMT on any safety concerns, and to contribute to recommendations regarding progression to a definitive RCT. As this is a small feasibility trial, a separate data monitoring committee has not been convened. The study sponsor is Guy’s and St. Thomas’ NHS Foundation Trust (GSTFT). The trial may be subject to audit by GSTFT under their remit as Sponsor.

### Progression to a full trial

This feasibility study will provide evidence of whether a definitive RCT could be undertaken. We will assess whether women with SCD are prepared to be recruited and randomised to a trial evaluating SPEBT, and we will collect information on outcomes important to participating women and sickle nurse specialists and associated care providers to inform the design of such a trial.

On completion of the feasibility study, we will use the analysis of outcomes to link to progression criteria, applying a traffic light system (green: proceed, yellow: consider changes, red: stop) as detailed in Table 2. Adequate recruitment, acceptability of the intervention, fidelity and reach, and retention will all be key to this assessment. Where there is discordance in the findings within the columns (for example, if we have green for the recruitment rate but yellow for dose and red for retention), then we will consider all available information, including the qualitative study findings to guide our decision. Throughout this process, we will work closely with our Expert PPI group, TSC and key stakeholders.

**Table 2:** ‘Traffic light’ criteria to assess progression to a full trial

	Recruitment rate of eligible participants (measuring reach, acceptability)	Frequency of SPEBT (measuring dose, acceptability)	Retention until 6 weeks post-delivery (measuring acceptability)	Findings from qualitative study and acceptability
Green	≥ 50%	≥ 75%, three or more	≥ 80%	Progress but will use findings from qualitative study to inform and improve definitive RCT
Yellow	30–50%	50–75%, three or more	50–80%	Use findings from qualitative study to inform progressing to definitive study depending on guidance from trial steering committee, Sickle Cell Society, PPI group and key stakeholders
Red	< 30%	< 50%, three or more	< 50%	Will not progress

### Dissemination plans

The trial results will be published in peer-reviewed scientific journals, and abstracts will be submitted to conferences. The study final report will be circulated to clinicians, service managers and commissioners through the RCOG, the British Society for Haematology, and NHS Clinical Commissioning Groups. A lay research summary will be disseminated to user groups such as the UK Haemoglobinopathy Forum and Sickle Cell Society, and to research participants and patient groups at collaborating hospitals.

## Discussion

Significant knowledge gaps exist on the effectiveness of SPEBT for pregnant women with SCD [9–11, 13]. This trial will allow us to assess whether women with SCD are prepared to be recruited and randomised to a definitive trial evaluating SPEBT and will provide crucial information on the acceptability of such a trial. This feasibility trial will enable us to collect information on outcomes important to participating women and sickle clinicians to inform the design of a definitive trial.

Practical issues for this trial include some elements of recruitment, particularly whether women will find it acceptable to be randomised to SPEBT. Although the proposed intervention is an invasive procedure with associated risks, our PPI work suggests that women with SCD who have experienced pregnancy feel that benefits from SPEBT may outweigh its risks. We note that successful studies of exchange transfusion have been conducted in other populations [17, 18]. Another practical issue for consideration is the potential difficulty in obtaining venous access in women with SCD; however, the participating centres all have well-established SPEBT programmes and staff who are highly skilled in obtaining peripheral venous access in this patient group. We are aware of the increased alloimmunisation in this patient group and will ensure that blood is matched for Rh and Kell blood groups.

The proposed study will provide important evidence about the feasibility of a definitive phase 3 trial of SPEBT in pregnant women, and the information collected through the trial and qualitative and economic sub-studies will be crucial in guiding the development of such a trial.

## Trial status

The protocol published here is version 1.1 dated 14 June 2019. The study began on 2 April 2019. Study recruitment commenced on 1 July 2019 and is expected to continue until 30 November 2020.

## Supplementary information

**Additional file 1.** SPIRIT 2013 Checklist: Recommended items to address in a clinical trial protocol and related documents.

## Data Availability

Not applicable.

## Abbreviations

CPMT: Core Project Management Team
Hb: haemoglobin
PPI: patient and public involvement
QALY: quality-adjusted life years
QoL: quality of life
RCOG: Royal College of Obstetricians and Gynaecologists
RCT: randomised controlled trial
SAP: statistical analysis plan
SCD: sickle cell disease
SPEBT: serial prophylactic exchange blood Transfusion
TSC: Trial Steering Committee
