# Supplementary material for: Serial prophylactic exchange blood transfusion in pregnant women with sickle cell disease (TAPS-2): study protocol for a randomised controlled feasibility trial
Source: Trials. 2020 Apr 20;21:347. doi: 10.1186/s13063-020-4212-8 (PMC7171865; doi:10.1186/s13063-020-4212-8)
Supplement: Supplementary file 1 — Additional file 1. SPIRIT 2013 Checklist: Recommended items to address in a clinical trial protocol and related documents. [file 13063_2020_4212_MOESM1_ESM.pdf]

SPIRIT
STANDARD PROTOCOL ITEMS: RECOMMENDATIONS FOR INTERVENTIONAL TRIALS

SPIRIT 2013 Checklist: Recommended items to address in a clinical trial protocol and related documents*

Section/item	Item No	Description	*Where addressed*
**Administrative information**			
Title	1	Descriptive title identifying the study design, population, interventions, and, if applicable, trial acronym	***Title.***
Trial registration	2a	Trial identifier and registry name. If not yet registered, name of intended registry	***At the end of abstract.***
	2b	All items from the World Health Organization Trial Registration Data Set	***Throughout.***
Protocol version	3	Date and version identifier	***In the "Trial status" section at the end of the main text (page 20).***
Funding	4	Sources and types of financial, material, and other support	***In the "Funding" section at the end of the main text (page 21).***
Roles and responsibilities	5a	Names, affiliations, and roles of protocol contributors	***Author list and the "Acknowledgments" section.***
	5b	Name and contact information for the trial sponsor	***In the "Sponsorship" section at the end of the main text (page 22).***
	5c	Role of study sponsor and funders, if any, in study design; collection, management, analysis, and interpretation of data; writing of the report; and the decision to submit the report for publication, including whether they will have ultimate authority over any of these activities	***In the respective sections ("Funding" and "Sponsorship").***

	5d	Composition, roles, and responsibilities of the coordinating centre, steering committee, endpoint adjudication committee, data management team, and other individuals or groups overseeing the trial, if applicable (see Item 21a for data monitoring committee)	***In the section "Trial governance and monitoring" (page 17). TSC membership is listed in the "Acknowledgements" section (page 22).***
**Introduction**			
Background and rationale	6a	Description of research question and justification for undertaking the trial, including summary of relevant studies (published and unpublished) examining benefits and harms for each intervention	***"Background" section (pages 4-7).***
	6b	Explanation for choice of comparators	***Methods section, under heading "Standard care" (page 9).***
Objectives	7	Specific objectives or hypotheses	***Beginning of "Methods" section (page 7). Also Table 1.***
Trial design	8	Description of trial design including type of trial (eg, parallel group, crossover, factorial, single group), allocation ratio, and framework (eg, superiority, equivalence, noninferiority, exploratory)	***Methods section (lines 155-157).***
**Methods: Participants, interventions, and outcomes**			
Study setting	9	Description of study settings (eg, community clinic, academic hospital) and list of countries where data will be collected. Reference to where list of study sites can be obtained	***Methods section ("Design and setting", page 7). A list of study sites is contained in the appendix.***
Eligibility criteria	10	Inclusion and exclusion criteria for participants. If applicable, eligibility criteria for study centres and individuals who will perform the interventions (eg, surgeons, psychotherapists)	***Methods section, under subheading "Participants" (page 7).***
Interventions	11a	Interventions for each group with sufficient detail to allow replication, including how and when they will be administered	***Methods section (pages 9-10).***
	11b	Criteria for discontinuing or modifying allocated interventions for a given trial participant (eg, drug dose change in response to harms, participant request, or improving/worsening disease)	***"Trial withdrawal or discontinuation" section (page 14).***

	11c	Strategies to improve adherence to intervention protocols, and any procedures for monitoring adherence (eg, drug tablet return, laboratory tests)	***n/a adherence to intervention can be assessed via clinical records***
	11d	Relevant concomitant care and interventions that are permitted or prohibited during the trial	***Methods section (page 9).***
Outcomes	12	Primary, secondary, and other outcomes, including the specific measurement variable (eg, systolic blood pressure), analysis metric (eg, change from baseline, final value, time to event), method of aggregation (eg, median, proportion), and time point for each outcome. Explanation of the clinical relevance of chosen efficacy and harm outcomes is strongly recommended	***Under "Outcomes" subheading (pages 11-12).***
Participant timeline	13	Time schedule of enrolment, interventions (including any run-ins and washouts), assessments, and visits for participants. A schematic diagram is highly recommended (see Figure)	***Figures 1 and 2.***
Sample size	14	Estimated number of participants needed to achieve study objectives and how it was determined, including clinical and statistical assumptions supporting any sample size calculations	***Under "Power and sample size" subheading (pages 14-15).***
Recruitment	15	Strategies for achieving adequate participant enrolment to reach target sample size	***Under "Power and sample size" subheading (pages 14-15).***
**Methods: Assignment of interventions (for controlled trials)**			
Allocation:			
Sequence generation	16a	Method of generating the allocation sequence (eg, computer-generated random numbers), and list of any factors for stratification. To reduce predictability of a random sequence, details of any planned restriction (eg, blocking) should be provided in a separate document that is unavailable to those who enrol participants or assign interventions	***Subheading "Randomisation" (pages 8-9).***
Allocation concealment mechanism	16b	Mechanism of implementing the allocation sequence (eg, central telephone; sequentially numbered, opaque, sealed envelopes), describing any steps to conceal the sequence until interventions are assigned	***Subheading "Randomisation" (pages 8-9).***
Implementation	16c	Who will generate the allocation sequence, who will enrol participants, and who will assign participants to interventions	***Subheading "Randomisation" (pages 8-9).***

Blinding (masking)	17a	Who will be blinded after assignment to interventions (eg, trial participants, care providers, outcome assessors, data analysts), and how	***Subheading "Randomisation" (pages 8-9).***
	17b	If blinded, circumstances under which unblinding is permissible, and procedure for revealing a participant's allocated intervention during the trial	***n/a trial is not blinded***
**Methods: Data collection, management, and analysis**			
Data collection methods	18a	Plans for assessment and collection of outcome, baseline, and other trial data, including any related processes to promote data quality (eg, duplicate measurements, training of assessors) and a description of study instruments (eg, questionnaires, laboratory tests) along with their reliability and validity, if known. Reference to where data collection forms can be found, if not in the protocol	***Section "data collection" (pages 10-11).***
	18b	Plans to promote participant retention and complete follow-up, including list of any outcome data to be collected for participants who discontinue or deviate from intervention protocols	***Section "data collection" (pages 10-11). Also "Trial withdrawal or discontinuation" section (page 14).***
Data management	19	Plans for data entry, coding, security, and storage, including any related processes to promote data quality (eg, double data entry; range checks for data values). Reference to where details of data management procedures can be found, if not in the protocol	***"Ethical considerations and confidentiality (page 16), "Data collection" (page 10).***
Statistical methods	20a	Statistical methods for analysing primary and secondary outcomes. Reference to where other details of the statistical analysis plan can be found, if not in the protocol	***Section "Analysis" (page 15).***
	20b	Methods for any additional analyses (eg, subgroup and adjusted analyses)	***Section "Analysis" (page 15).***
	20c	Definition of analysis population relating to protocol non-adherence (eg, as randomised analysis), and any statistical methods to handle missing data (eg, multiple imputation)	***Section "Analysis" (page 15).***
**Methods: Monitoring**			

Data monitoring	21a	Composition of data monitoring committee (DMC); summary of its role and reporting structure; statement of whether it is independent from the sponsor and competing interests; and reference to where further details about its charter can be found, if not in the protocol. Alternatively, an explanation of why a DMC is not needed	***In the section "Trial governance and monitoring" (page 17) (n.b. no DMC to be convened).***
	21b	Description of any interim analyses and stopping guidelines, including who will have access to these interim results and make the final decision to terminate the trial	***n/a (no DMC and small feasibility trial only)***
Harms	22	Plans for collecting, assessing, reporting, and managing solicited and spontaneously reported adverse events and other unintended effects of trial interventions or trial conduct	***"Ethical considerations and confidentiality" (pages 16-17).***
Auditing	23	Frequency and procedures for auditing trial conduct, if any, and whether the process will be independent from investigators and the sponsor	***In the section "Trial governance and monitoring" (page 17)***
**Ethics and dissemination**			
Research ethics approval	24	Plans for seeking research ethics committee/institutional review board (REC/IRB) approval	***Ethics approval received, details given under "Ethics approval and consent to participate" (page 20).***
Protocol amendments	25	Plans for communicating important protocol modifications (eg, changes to eligibility criteria, outcomes, analyses) to relevant parties (eg, investigators, REC/IRBs, trial participants, trial registries, journals, regulators)	***In the section "Trial governance and monitoring" (page 17).***
Consent or assent	26a	Who will obtain informed consent or assent from potential trial participants or authorised surrogates, and how (see Item 32)	***Subheading "Participant identification and recruitment" (page 8).***
	26b	Additional consent provisions for collection and use of participant data and biological specimens in ancillary studies, if applicable	***n/a no ancillary studies planned***
Confidentiality	27	How personal information about potential and enrolled participants will be collected, shared, and maintained in order to protect confidentiality before, during, and after the trial	***"Ethical considerations and confidentiality" (page 16).***

Declaration of interests	28	Financial and other competing interests for principal investigators for the overall trial and each study site	***"Competing interests" (page 21).***
Access to data	29	Statement of who will have access to the final trial dataset, and disclosure of contractual agreements that limit such access for investigators	***"Data collection" (page 10).***
Ancillary and post-trial care	30	Provisions, if any, for ancillary and post-trial care, and for compensation to those who suffer harm from trial participation	***None.***
Dissemination policy	31a	Plans for investigators and sponsor to communicate trial results to participants, healthcare professionals, the public, and other relevant groups (eg, via publication, reporting in results databases, or other data sharing arrangements), including any publication restrictions	***"Dissemination plans", (page 18).***
	31b	Authorship eligibility guidelines and any intended use of professional writers	***We will follow established guidelines for deciding authorship. No use of professional writers is planned.***
	31c	Plans, if any, for granting public access to the full protocol, participant-level dataset, and statistical code	***Not yet confirmed.***
**Appendices**			
Informed consent materials	32	Model consent form and other related documentation given to participants and authorised surrogates	***Available on request.***
Biological specimens	33	Plans for collection, laboratory evaluation, and storage of biological specimens for genetic or molecular analysis in the current trial and for future use in ancillary studies, if applicable	***n/a no biological specimens to be collected***

*It is strongly recommended that this checklist be read in conjunction with the SPIRIT 2013 Explanation & Elaboration for important clarification on the items. Amendments to the protocol should be tracked and dated. The SPIRIT checklist is copyrighted by the SPIRIT Group under the Creative Commons "Attribution-NonCommercial-NoDerivs 3.0 Unported" license.
